# Bank erosion drastically reduces oyster reef filtration services in estuarine environments

**DOI:** 10.1038/s41598-024-66670-1

**Published:** 2024-07-09

**Authors:** Daniele Pinton, Alberto Canestrelli

**Affiliations:** Department of Civil and Coastal Engineering, University of Florida, Gainesville, FL USA

**Keywords:** Bank erosion, Oyster reefs, Filtration services, Numerical modeling, Environmental sciences, Ocean sciences

## Abstract

Oyster reefs near estuarine channels have experienced substantial mortality over the last decades, primarily due to bank erosion, potentially exacerbated by boat activity. Using aerial imagery, we measured bank erosion along the Intracoastal Waterway and its main tributaries in the Guana-Tolomato-Matanzas estuary, finding that erosion outweighs progradation. This notably threatens oyster reefs and their filtration capabilities. By modeling the impact of bank erosion on oyster habitats and filtration using hydrodynamic, water quality, and particle tracking models, we observed a 12% filtration reduction due to reef mortality. Erosion results in an exponential decrease in reef area and filtration services, due to the removal of channel-adjacent reefs, which play a critical role in water filtration. If current erosion rates continue, simulations suggest a potential 20% filtration reduction over 100 years, potentially worsening water quality. Our findings highlight the urgency to protect and restore reefs near banks to mitigate erosion and maintain filtration services.

## Introduction

Oyster reefs stand out as crucial contributors to the health and stability of estuarine environments. They offer essential protective benefits such as wave dissipation, shoreline erosion reduction, and an increase in sediment deposition^1–3^. Also, they provide non-protective services, by supporting aquatic food webs^2,4^ and offering recreational opportunities^5^. Additionally, oyster reefs have recently gained increased attention for their resilience and self-maintenance properties in a changing climate^6,7^, becoming an attractive nature-based alternative to anthropogenic infrastructure. Oyster reefs are also renowned for their ability to filter vast volumes of water, removing particulate matter and pollutants suspended in the water column^8^. This filtration capacity not only improves water clarity^9^ but also enhances the overall quality of estuarine environments by mitigating nutrient loading and facilitating denitrification^10^.

Despite their ecological significance, oyster reefs face numerous threats causing the decline in both their area and the populations of oysters that inhabit them. Changes in water quality^11,12^, diseases, predation^13,14^, and anthropogenic activities such as overharvesting^14–17^ are primary drivers of reef reduction.

Bank erosion and oyster reef habitat loss have been linked to similar processes and stressors. Many studies have shown a clear link between the increase in boating activity and oyster reef mortality^18–22^. Along the Intracoastal Waterway (ICW) and near the Guana-Tolomato-Matanzas (GTM) estuary, oyster reef habitat loss and bank erosion have been consistently linked to the increase in boat traffic^18,19,22,23^. Additionally, Garvis et al.^22^ suggest that the mortality of oyster reefs and the subsequent formation of shell rakes can contribute to coastal erosion. Oyster reefs are known to protect marsh and river banks. However, over a certain activity threshold, boat wakes erode sediment around *Crassostrea virginica* reefs and cause them to be displaced onto the top of the reef platform or potentially on top of an adjacent marsh where they perish due to an increase in aerial exposure, and a decrease in both feeding and larval settlement^20,24^. This process creates dead reefs composed of disarticulated shells (i.e., dead margins). Studies have observed dead reefs moving away from boating channels at a rate of 0.86 ± 0.14 m year^−1^^24^. Boat wakes additionally contribute to sediment resuspension within the channel and subsequent deposition onto nearby reefs, leading to their gradual burial and eventual death^18^. While wind contributes to wave generation, the occurrence of dead margins specifically around boating channels, their absence in wind-exposed areas, and the limited impact of storms on oyster reef structure, observed in the field, indicate that boat activity is the main driver of reef degradation^18,20,22^.

The decline of oyster reefs not only diminishes habitat availability but also reduces their filtration services, which can result in decreased water quality and an impaired ecosystem^25^. The loss of oyster reef filtration services can have cascading effects on estuarine ecosystems, leading to increased turbidity, nutrient enrichment, and altered species composition. Additionally, the decline in water filtration can lead to more frequent and severe outbreaks of illnesses linked to poor water conditions^26,27^. This can also have adverse economic impacts. The fisheries sector, which depends on clean water for healthy fish stocks, could collapse^28^, endangering the livelihoods of many who depend on fishing. Similarly, the tourism industry, a substantial economic driver in the GTM estuary, can be impacted. In particular, tourists can be deterred by the compromised water conditions, leading to a decline in recreational water activities and waterfront visits^29,30^.

To quantify the filtration capacity of oyster reefs and assess their contributions to water quality improvement, researchers have developed various models^31–33^. These models integrate factors such as oyster density, reef morphology, hydrodynamic conditions, and water quality parameters to estimate filtration services and the removal of particulate matter and pollutants from the water column. For instance, Lagrangian methods are widely used to model the motion of general pollutants in waterbodies^34–36^. In these models, pollutants are treated as particles whose movement is driven by local hydrodynamics, such as tides and currents. As models advance in complexity, there has been a growing emphasis on incorporating realistic ecological and morphological processes for the interaction of oyster reefs with their environment^37,38^.

**Figure 1:**
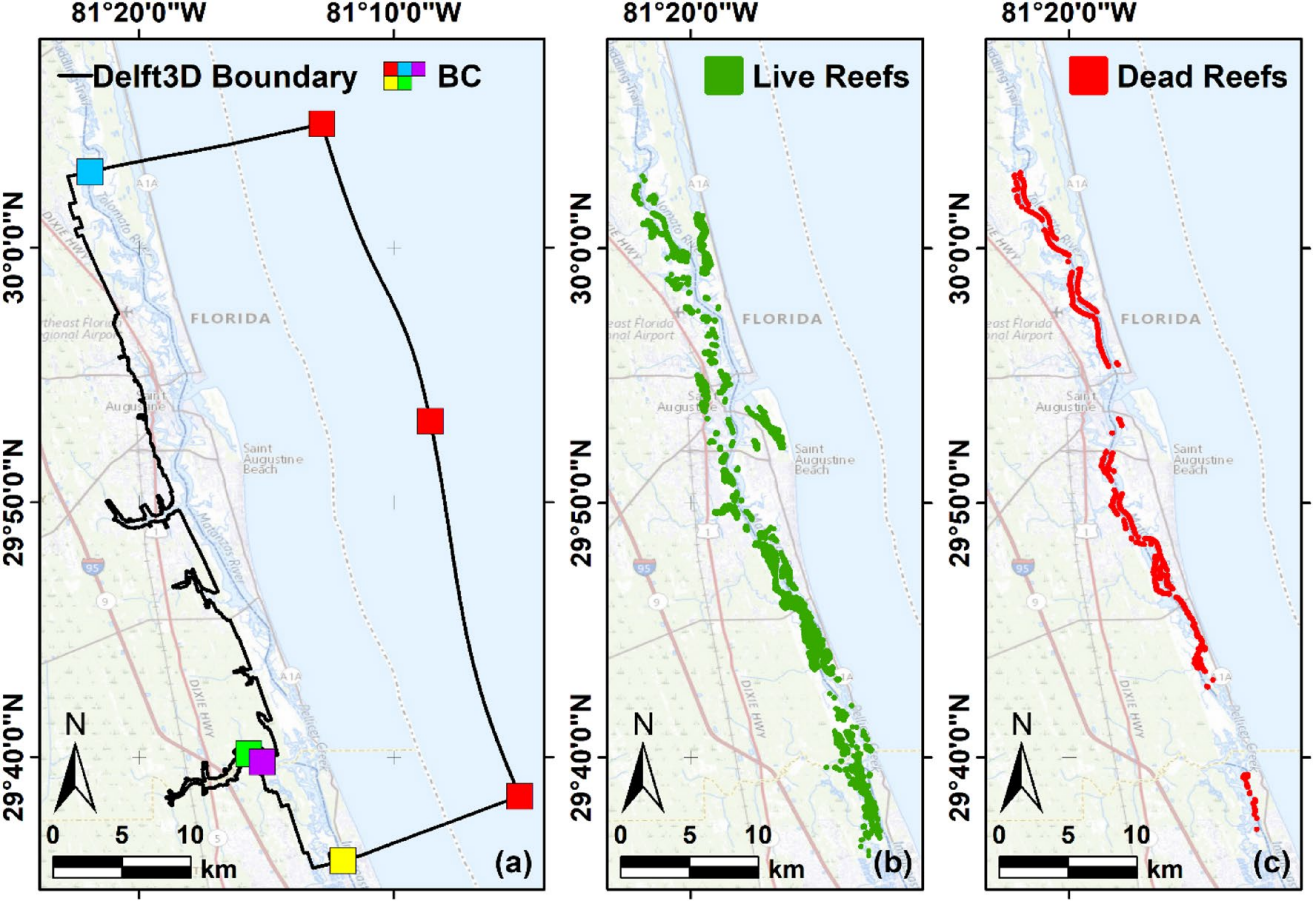
Study area and oyster reefs distribution. (**a**) The Guana-Tolomato-Matanzas estuary. The black line is the Delft3D model domain boundary. The colored squares are the boundary conditions of the model. The distribution of the oyster reefs with living (**b**) and dead (**c**) oyster populations along the model domain, according to the FWC Florida oyster beds database (Maps generated using ESRI ArcGIS, v.10.8.1, https://www.arcgis.com/index.html. Background: ESRI World Ocean base).

In this paper, we incorporate bank erosion estimates, oyster reef distribution, hydrodynamic processes, and oyster reef filtration services in a numerical model describing an estuarine environment. Filtration services are calculated by coupling a hydrodynamic and particle tracking numerical model (Delft3D) with an oyster filtration model (MATLAB). Note that, we do not focus on a specific type of particle. Instead, we consider a general pollutant moving in our study area due to local hydrodynamic forces, which can be retained by oysters during the filtration process. Our study is centered on the GTM estuary, located in Northeastern Florida (see Fig. 1a), and intersected by the ICW via the Matanzas and Tolomato Rivers. We selected this estuary due to its rich oyster reef populations, historical trends of boat-driven bank erosion in its main rivers since 1970^23^, and the presence of deteriorated oyster reefs along the ICW. Our findings highlight the substantial impact of boat wave-driven bank erosion on water quality in estuaries, which can potentially reduce the filtration efficiency of oyster reefs in the short term. These findings are crucial for guiding conservation and management strategies aimed at preserving these essential ecosystems.

## Results

### Bank erosion

To evaluate the potential impact of boat traffic on bank erosion, we calculate bank lateral movement in the GTM estuary by analyzing airborne images extracted from the NOAA imagery inventory (https://coast.noaa.gov/dataviewer/#/). These include three RGB datasets collected by USGS (2006) and NOAA (2015 and 2023). We indicate the negative and positive values of calculated bank lateral movement as erosion and progradation, respectively.

**Figure 2:**
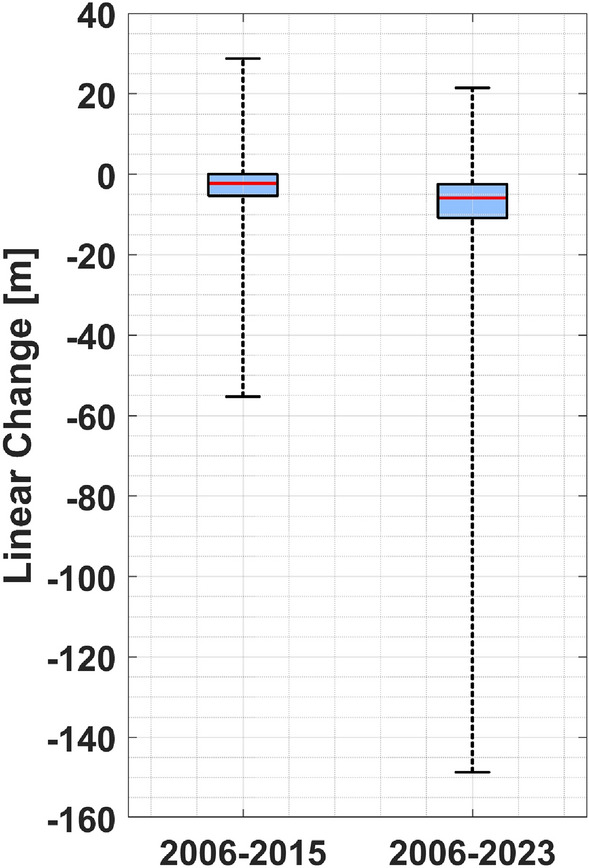
Bank erosion estimate. The lateral movement of banks in the ICW measured in the periods 2006–2015 and 2006–2023 from historical airborne images.

We observe that lateral movement in bank position in the GTM estuary between 2006 and 2015 ranges from − 60 to + 30 m, with an average value of − 3.30 m (first column in Fig. 2). The average lateral movement rate is approximately − 0.30 ± 0.93 (mean ± standard deviation) m per year. In addition, between 2006 and 2023, lateral movement in the bank ranged from − 150 m to + 60 m, with an average value of − 8.30 m (second column in Fig. 2) and an average annual migration rate of about − 0.50 ± 0.74 m per year.

### Present-day filtration scenario

We use the Delft3D-FLOW + PART model coupled with an oyster filtration model^31^ to evaluate oyster filtration in different oyster distribution scenarios. The location and extent of oyster reefs in the GTM estuary were determined using the Florida oyster beds database (https://hub.arcgis.com/datasets/myfwc::oyster-beds-in-florida) from the Fish and Wildlife Conservation Commission (FWC). This database contains approximately 5200 reefs classified into two groups: alive (~ 4800, Fig. 1b) and dead (~ 400, Fig. 1c).

**Figure 3:**
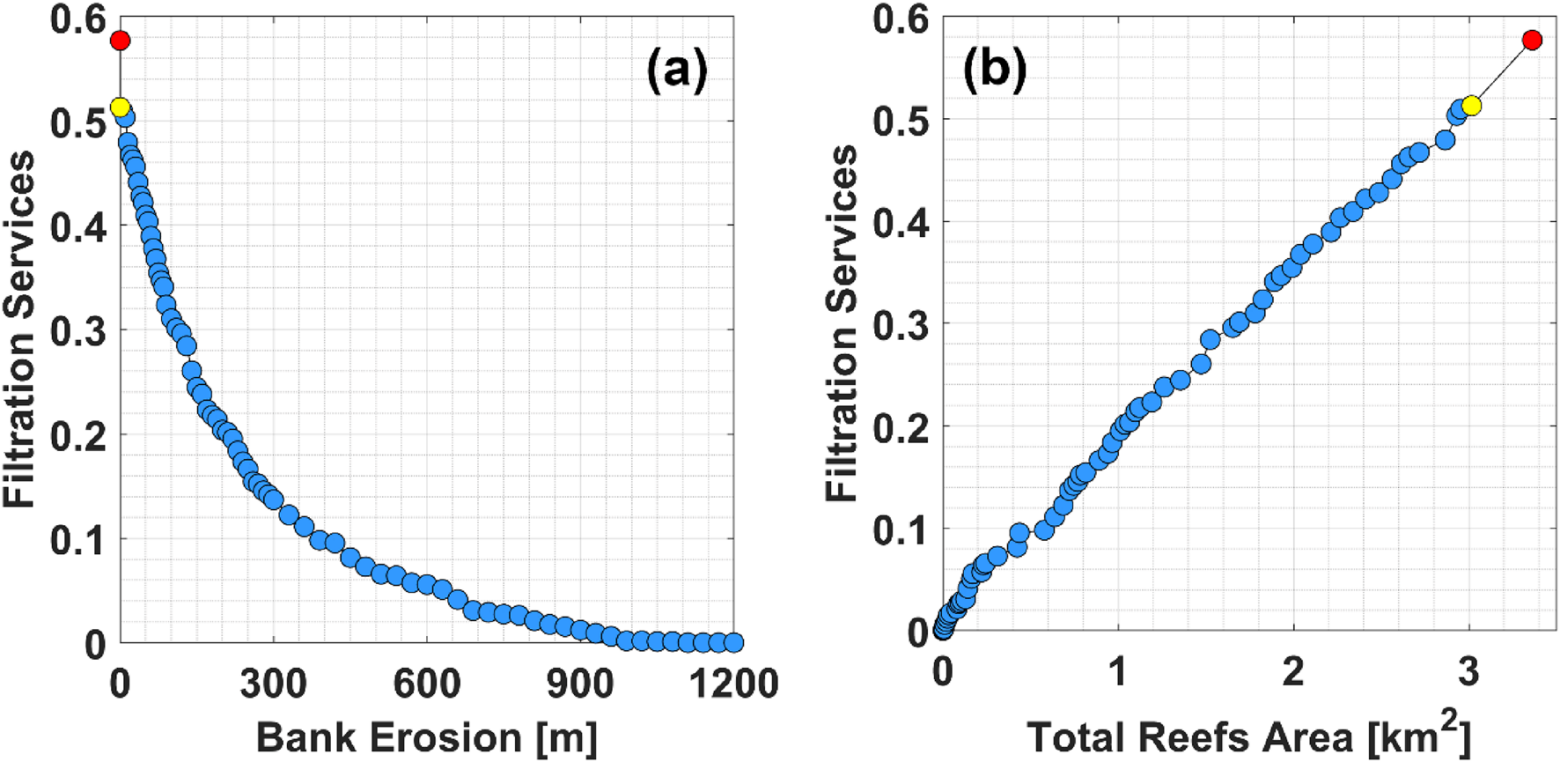
Filtration services vs. bank erosion, and reefs area. (**a**) $FS$ vs. bank erosion. (**b**) $FS$ vs. total oyster reefs area. Blue dots: Declining estuary-scale filtration due to oyster reef removal along distances from the bank (0 m to 1200 m), in 30 m intervals, to simulate bank erosion in the GTM estuary. Yellow dots: Current scenario with filtration only from reefs with live oyster populations. Red dots: Pre-mortality scenario with filtration from reefs with both live and dead oyster populations.

**Figure 4:**
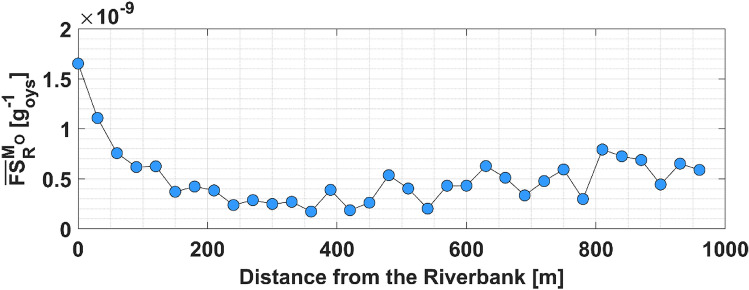
${\overline{FS} }_{R}^{{M}_{O}}$ vs. distance from the riverbank. The average value of Filtration Services per unit of oyster mass (${\overline{FS} }_{R}^{{M}_{O}}$) calculated for reefs located at various distances from the banks in the GTM estuary, in the scenario with filtration from reefs with live oyster populations.

**Figure 5:**
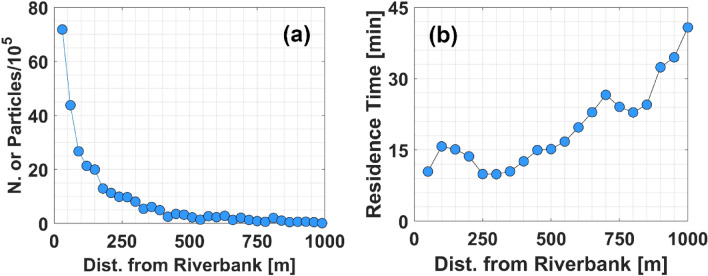
Particle dynamics and residence time. (**a**) Number of particles entering the oyster reefs placed at increasing distances from the banks. (**b**) Average residence time calculated for reefs placed at increasing distances from the banks.

In the present-day scenario, only oyster reefs populated by live oysters contribute to water filtration. These reefs play a crucial role in purifying water in estuaries, filtering approximately 51% of pollutants during an estuary residence time (yellow dots in Fig. 3), which is equal to almost 13 days^31,39^. Note that, in Gray et al.^31^, we define the estuary residence time as the time needed for the particles injected in the GTM estuary to decrease their number by 1/$e$ (with $e$≈2.7). Reefs located close to the banks show higher filtration services per unit of oyster mass ($F{S}_{R}^{{M}_{O}}$, see Fig. 4). The filtration capacity of a reef increases with (i) the reef area (${A}_{R}$), (ii) the reef oyster density (${D}_{O}$), and (iii) the mass (dry tissue weight, $DTW$) of the average oyster populating the reef, which depends on its size (see Eqs. (2)–(4)). The total mass of the oysters populating a reef (${M}_{O}$) is calculated as ${M}_{O}={A}_{R}{D}_{O}DTW$. Thus, we use $F{S}_{R}^{{M}_{O}}$ to determine the individual impact of each reef on estuarine filtration services, regardless of their area, oyster density, and oyster size. On average, the value of $F{S}_{R}^{{M}_{O}}$ gradually decreases as reefs are placed farther from the banks (Fig. 4). Note that at 60 m from the bank the value of $F{S}_{R}^{{M}_{O}}$ drops to half of what is observed for reefs placed at the bank. The lowest $F{S}_{R}^{{M}_{O}}$ is observed at 350–400 m from the bank, before gradually increasing again reaching almost half of the value observed close to the rivers for reefs located 900–1000 m from the ICW. These reefs have a higher value of ${FS}_{R}^{{M}_{O}}$ compared to those located at intermediate distances from the bank due to the higher value of residence time ($RT$) observed in the area they occupy (Fig. 5b). The lower value of ${FS}_{R}^{{M}_{O}}$ observed for reefs located at intermediate distances from the bank is due to the concomitance of reduced $RT$ and a lower passage of particles above them.

### Pre-mortality filtration scenario

In the pre-mortality scenario, oyster reefs labeled as "dead" in the FWC dataset are occupied by live oyster populations, thereby contributing to the filtration of GTM estuary water. This scenario simulates conditions before the oyster populations above those reefs became extinct. In this scenario, the filtration services provided at the estuary scale are approximately 0.58 (red dots in Fig. 3). This suggests that the mortality of oyster reefs, likely caused by the rise in boating activity in the GTM, results in a decline in filtration services by 0.07, which corresponds to a 12% decline in percentage terms.

### Future estimate of filtration services reduction due to bank erosion

Filtration services are predicted to reduce exponentially as bank erosion increases, and the area occupied by oyster reefs decreases (blue dots in Fig. 3a). This relationship is described by the equation (R^2^ = 0.997):

1 $$FS=0.508{e}^{-0.0044{E}_{B}},$$

where ${E}_{B}$ is the bank erosion in meters. Note that these rates have not been computed by simply removing the filtration of the eroded reef. Rather, they are recomputed by re-running the filtration module every time reefs are removed from the model domain. This procedure ensures that spurious “downstream effects” are removed^31^, i.e. that removed reefs do not influence the filtration rates of adjacent reefs. Note that the $FS$ provided by the oyster reefs in the GTM estuary reduces by ~ 50% when bank erosion reaches 150 m. Also, note that $FS$ becomes almost null when bank erosion reaches approximately 950 m. This suggests that reefs growing beyond 950 m from the ICW have a negligible impact on estuarine water filtration. Also, the area occupied by the oyster reefs decreases exponentially with bank erosion, since most of the reefs are localized close to or at the bank. For instance, about half of them are within 100 m of the bank. For this reason, filtration services in the estuary reduce almost linearly with a decrease in reef area due to bank erosion (Fig. 3b). Finally, note that the decrease in $FS$ resulting from the removal of dead oyster reefs (red dot) follows the exponential and linear trend observed in Fig. 3a,b, respectively.

## Discussion

### Bank erosion

Our findings indicate the predominance of bank erosion over progradation in both analyzed periods (i.e., 2006–2015 and 2006–2023), an increase in the magnitude and range of bank erosion, and a decrease in the magnitude and range of bank progradation over time, in the GTM estuary. This is confirmed by the results shown in Fig. S1 of the supplementary material. Analysis of imagery datasets suggests that progradation is primarily due to the presence of mangroves and the progressive increase in their habitat area in the GTM estuary in response to environmental warming^40^. The absence of an increase in oyster reef habitats in areas where mangrove habitats are expanding can be attributed to competition between these two species and the inability of mangroves to create suitable settling substrates for oysters in short periods. The results also indicate a progressive shift from a mixed regime of progradation and bank erosion to a regime of only erosion over time along the GTM estuary. Previous analysis of bank erosion in the GTM estuary shows an average bank migration rate of approximately − 0.30 m per year^23^. This rate aligns with our calculations from 2006 to 2015 but is lower than the average observed between 2006 and 2023, which was − 0.50 m per year. This suggests a non-linear trend in bank erosion increase within the GTM estuary. The analysis of horizontal accuracy of imagery datasets and systematic error in detecting marsh edges shows that bank erosion predominates over progradation. Detailed results are available in the supplementary material (section “Error Analysis on Bank Erosion Estimates”) .

Bank erosion is often linked to factors such as sea level rise (SLR)^41,42^, storm-generated wind waves^43^, and human activities^44^. While SLR is a contributing factor, data from NOAA stations (i.e., those listed in Table 1) show no substantial increase in SLR rates in our study area. In addition, a recent study indicated that the marshes in the GTM National Estuarine Research Reserve (GTMNERR) have demonstrated resilience, maintaining their productivity and keeping up with rising sea levels^45^. Also, although large storms play a minor role, accounting for less than 1% of long-term salt marsh lateral erosion rates, the degradation of salt marshes is primarily driven by average wave conditions^43^. This phenomenon occurs because of the linear relationship between wave power and erosion rate, coupled with the brief duration of extreme events^43^. Although moderate weather conditions prevail for most of the year, the erosion potential of extreme events is concentrated within just a few days each year. Moreover, in systems with small inlets, extensive barrier islands, and small fetch such the GTM, even during hurricane conditions the wave conditions are small^46^. Wind waves can also play a role in bank erosion. However, their impact on bank erosion is typically smaller than that of boat waves due to the limited width and consequently the limited fetch of the channels in the ICW, where wind waves are generated. Additionally, due to the narrow channel width, the ICW does not provide a significant distance for wake energy to subside before impacting its margins^46^. Additionally, Theuerkauf et al.^47^ suggest that boat waves play a substantial contribution in shoreline erosion. Finally, Fonseca and Malhotra^48^ indicates that approximately 50% of boat wake heights surpass the largest wind-generated waves in the Intracoastal Waterway in North Carolina. This suggests that boat wakes likely exceed the wind wave background in many areas of the Intracoastal Waterway. Therefore, the observed increase in bank erosion is more likely attributable to heightened boat traffic along the ICW. This aligns with previous studies indicating that boat wakes play a substantial role in bank erosion and the deterioration of oyster reefs along the ICW^49,50^. In addition, note that the combination of increased bank erosion, boating activity, and SLR can lead to the replacement of salt marshes with tidal flats, altering local flow patterns and increasing water fluxes, bed shear stress, and sediment resuspension^51,52^ in the GTM estuary. These processes can negatively impact the resistance and growth of oyster reefs by increasing mortality due to burial, reducing larvae settlement, and limiting their feeding activity^18,53^. Furthermore, bank erosion can amplify fetch values and contribute to the generation of higher waves, potentially exacerbating erosion along the banks. This process can accelerate the degradation of oyster reef habitats and their filtration services.

**Table 1:** Available stations and datasets used for the boundary conditions in the Delft3D numerical model.

Section ID	Station name	Agency	Latitude longitude	Data
8,720,291	Jacksonville Beach, FL	NOAA	30° 17′ 00″ N 81° 23′ 12″ W	Harmonic constituents
8,720,587	St. Augustine Beach, FL	NOAA	29° 51′ 24″ N 81° 15′ 48″ W	Harmonic constituents
8,721,020	Daytona Beach (ocean), FL	NOAA	29° 13′ 42″ N 81° 00′ 18″ W	Harmonic constituents
GTMPIWQ	Pine Island	NERR	30° 03′ 03″ N 81° 22′ 03″ W	Water temperature and water level
GTMPCWQ	Pellicer Creek	NERR	29° 40′ 01″ N 81°15′27″ W	Water temperature
GTMPCMET	Pellicer Creek	NERR	29° 39′ 28″ N 81° 13′ 58″ W	Meteorological data
02,247,222	Pellicer Creek near Espanola, FL	USGS	29° 40′ 09″ N 81° 15′ 35″ W	River discharge
8,720,757	Bings landing, Matanzas river, FL	FDEP	29° 36′ 54″ N 81° 12′ 18″ W	Water temperature and water level

Additionally, significant changes due to bank erosion, such as altered substrates, reduced water quality, and increased water depth, can severely impact the formation and survival of new natural and restored oyster reefs in these areas. An analysis of imagery datasets and FWC oyster bed datasets reveals that no reefs have developed along the ICW in front of dead reefs. Instead, the images indicate that local oyster racks, which formed due to reef death and could have served as habitats for oyster larvae, have been completely eroded or retreated towards the mainland. This demonstrates that oyster reefs cannot sustain themselves in areas affected by bank erosion, in the GTM estuary.

Finally, the decline in oyster reef habitats could have substantial economic repercussions for the local economy by jeopardizing fisheries and tourism, which in turn may lead to fewer job opportunities. Kroeger^54^ estimated that restoring 100 miles of oyster reef in Mobile Bay, AL, USA, could boost the local economy by approximately $18.8 million annually and create about 204 jobs each year. Extrapolating these data to our study area, the documented loss of 3,500,000 m^2^ oyster reefs in the GTM, corresponding to ~ 25 miles, could be linked to an annual economic loss of around $4.7 million and an annual reduction in job opportunities of about 51. Projecting these losses over 100 years with an estimated ~ 200,000,000 m^2^ of oyster reef loss, corresponding to 97 miles, the economic impact could reach approximately $18.14 million per year, with about 197 jobs lost annually. Similar results are obtained by using the estimates in Grabowski et al.^5^, who suggested that the economic value of oyster reef services, excluding oyster harvesting, is between $5500 and $99,000 per hectare per year.

### Filtration services reduction because of bank erosion

In the GTM estuary, the river network acts as a conduit for all particles released into the estuary. During flood tide, these particles are dispersed by the tide to marshes and tidal flats, which are bordered or occupied by reefs. Being in this strategic position, these reefs are the first to intercept pollutants coming from the ICW. Consequently, they filter a greater quantity of pollutants compared to reefs situated farther from the bank, as they receive particles already filtered by upstream reefs. In addition, during both flood and ebb tides, these reefs intercept and filter all particles leaving the salt marshes and tidal flats before they reenter the ICW, and vice versa. Therefore, these reefs present the highest $F{S}_{R}^{{M}_{O}}$, making them key participants in estuarine water filtration.

However, these reefs are also most threatened by bank erosion, and they are likely to be the first to disappear due to the exacerbation of this phenomenon. Moreover, note that their filtration services are only partially transferred to surviving reefs. The influx of particles into reefs decreases with increasing distance from the bank (Fig. 5), Consequently, in the event of reef mortality leading to a cessation of particle filtration, there is no guarantee that a distant reef situated beyond the deceased one will effectively filter those particles. Our results suggest that a 20% reduction in filtration services could occur within 100 years in the GTM estuary at the current average rate of bank erosion. Note that, for this analysis, all factors potentially affecting hydrodynamics and bank erosion, such as wind waves, tides, turbidity of the estuary, and boat traffic, are assumed to remain constant and are based on the average conditions observed between 2006 and 2023, in which we calculated the rate of bank erosion observed along the GTM estuary. We assume this to be a conservative estimate for boat wakes, given the continuous increase in boating activity measured in the estuary (Vessel traffic database: https://marinecadastre.gov/ais/; Dr. Nikki Dix, GTMNERR, personal communication). The observed trend of increasing average bank erosion could hasten this scenario. Furthermore, our analysis reveals peaks of 150 m in bank erosion over the past 20 years, highlighting the potential of bank erosion to endanger oyster reefs even in shorter periods. Accordingly, similar reductions (24–40%) in oyster habitat areas have been observed in the nearby Mosquito Lagoon and Canaveral National Seashore over 60 years, associated with an increase in boat traffic^19,20,22^. Additionally, our results confirm the negligible importance of pollutant particles water residence time compared to their encounter rate with oyster reefs in estuarine filtration services^31^. This suggests that restoring or protecting oyster reefs by prioritizing locations with high residence time^32^ may not be the optimal approach. Finally, note that our approach is general. Let us assume we have given a certain mass ($M$) of pollutants in the estuary, and our model estimates that bank erosion reduces filtration services provided by the oyster reefs by $K$%. Since it has been shown that filtration is proportional to the concentration of pollutants in the water column (Eq. (4), see also Cerco and Noel^55^), if pollution increases by $N$ times, the mass entering the estuary is $NM$, and reduction in filtration services is still $K$% of $NM$.

Temperature significantly influences oyster reef filtration services. Our results already account for daily fluctuations in $FS$ by using simulated temperatures from the study period, which represents the average spring + neap tides of the year. The average water temperature during this period closely matches the yearly average, making our calculations of $FS$ representative of its annual average.

In conclusion, our study underscores the critical importance of monitoring and managing boat traffic near oyster reef habitats, particularly in estuarine channels like the Guana-Tolomato-Matanzas estuary. The substantial mortality of oyster reefs over the past few decades, primarily attributed to channel bank erosion exacerbated by boat activity, poses a severe threat to water quality and ecological balance in these sensitive ecosystems. Our findings suggest a pressing need for collaborative efforts among stakeholders to initiate discussions and implement strategies aimed at mitigating the impact of boat traffic on oyster reef habitats. By protecting and restoring reefs near banks, we can address erosion, sustain filtration services, and ultimately safeguard the health and resilience of estuarine ecosystems for future generations.

## Methods

### Bank erosion

To calculate bank erosion: (i) We identify 100 areas along the ICW. These areas are uniformly distributed across the study region and present in all three RGB datasets. (ii) We manually identify the bank in each RGB dataset by defining it as the limit of vegetated areas close to the river (see Fig. S2a–c, in the supplementary material). (iii) We wrote a MATLAB script to measure the distance between the banks and the river axes (see Fig. S2d, in the supplementary material). This script divides the axes into 50-m segments. At the end of each segment, it draws a straight line perpendicular to the river axes, intersecting the banks. It then computes the distance between these intersection points and the river axes in all three RGB datasets. This approach closely resembles the one used in Price^23^ to evaluate bank erosion in the same area between 1970 and 2002. (iv) Bank erosion is calculated as the difference between the distances determined in the previous step.

### Hydrodynamic model details

We use the Delft3D-FLOW model^56^ to solve the hydrodynamics and the temperature exchange in the GTM estuary. Note that, in the GTM estuary, hydrodynamic is mainly driven by the tide^31,39^. The model calculates non-steady flow resulting from the tidal and meteorological forcing on a regular, boundary-fitted grid. The Delft3D model domain, shown in Fig. 1a (black line), encompasses the GTMNERR and is centered on the city of St. Augustine, FL, USA. The grid cell size ranges from approximately 30 m × 100 m in the ocean to around 15 m × 20 m in the estuary. The bathymetric data for the ocean in the model are extracted from the NOAA inventory. For the GTM, bathymetry incorporates information from various sources. These include the Florida Natural Areas Inventory vegetation map, and the topographic and bathymetric Lidar data from the USGS, USACE, and NOAA.

### Delft3D model scenario

Our simulation goes from May 9th, 2018, to June 10th, 2018. This period, calculated as in Gray et al.^31^ and Crotty et al.^57^, contains approximately 2 neap tides and 2 spring tides, which are the most representative of the year^31^. For the simulation, we use a time step of one minute. As boundary conditions we apply: (i) the harmonic constituents of the astronomical tide, obtained from three local NOAA stations, and the water temperature extrapolated from the Navy Coastal Ocean Model at the offshore boundary (red squares in Fig. 1a), (ii) the water level and water temperature data obtained from the GTMNERR and FDEP stations, at the northern and southern boundaries of the ICW, respectively (blue and yellow squares in Fig. 1a), (iii) the tidally filtered discharge measured at the USGS station at Pellicer Creek (green square in Fig. 1a), and (iv) the meteorological forcings, corresponding to relative humidity, air temperature, wind direction, wind speed, precipitation, and solar radiation, across the entire domain recorded at the meteorological station at Pellicer Creek (purple square in Fig. 1a). Information about the stations we use to get data for the model, and about the extracted time series, are reported in Table 1. Finally, to verify the impact of roughness values on filtration rates, we conducted a simulation where we adjusted oyster reef friction by ± 20%. The results showed less than a 5% change in average velocity above the reefs and less than a 1% change in average velocity across the entire estuary. Given that filtration depends more on local hydrodynamics than residence time above the reefs^31^, these findings indicate that oyster reef roughness does not significantly affect filtration rates at the estuary scale.

### Oyster reefs data

Between 2014 and 2023, the GTMNERR conducted surveys to collect data on oyster populations in the GTM estuary. This included metrics like shell height and oyster density across 250 reefs. To assess oyster density (${D}_{O}$) and oyster shell height ($SH$) across the entire estuary, we utilize an inverse distance weighted interpolation method using MATLAB (Release 2023b) on the data collected from these reefs^31^.

### Allometric function

Population models commonly employ the allometric function $B=a{L}^{b}$ to link a linear dimension $L$ (e.g., $SH$) to an energy determinant $B$ (e.g., dry tissue weight, $DTW$). Fitting the data from Gray et al.^31^ with this function, we define the correlation between $DTW$ (in grams) and $SH$ (in mm) by using the following equation:

2 $$DTW=0.00014685 {SH}^{1.9473}.$$

The data from Gray et al.^31^ with the fitting line from Eq. (2) are shown in Fig. S3 in the supplementary material.

### Physiology

The oyster filtration rate (${FR}_{O}$) is defined as the volume of seawater filtered per unit of time by each single oyster (m^3^h^-1^oyster^-1^). In this paper, ${FR}_{O}$ is defined as follows:

3 $${FR}_{O}=8.02{ DTW}^{0.58}{e}^{{-0.015\left(T-27\right)}^{2}},$$

where $T$ is the water temperature (in °C) above the reef. Equation (3) is presented in Fig. S4 in the supplementary material, for the dry mass ($DTW$) of the average oyster in the GTM estuary. Equation (3) is based on the approach zu Ermgassen et al.^25^ employed to examine the services of individual oysters along the Atlantic and Gulf Coast, modified as proposed by Cerco and Noel^55^ to incorporate the effect of temperature on filtration. The filtration rate of a reef ($F{R}_{R}$) is the sum of the individual filtration rates (${FR}_{O}$) of all the oysters within the reef. The number of oysters is calculated by multiplying the area of the reef (${A}_{R}$) by the oyster density on the reef (${D}_{O}$).

### Filtration services calculation

Oyster $FS$ represents the proportion of water filtered in the estuary over one residence time. To assess the individual contributions of different reefs to the overall filtration services in the estuary, we use the MATLAB code proposed in Gray et al.^31^. This code simulates interactions between virtual oysters and suspended particles using Delft3D-PART. We simulate six releases of particles into the estuary, spaced two hours apart, covering a full tidal cycle to account for tidal effects. One particle is injected in each cell at each release. Injection points are located at the midpoints of 50 m × 50 m grid cells, representing the wetted area of each watershed. The particle tracking simulation uses a one-minute time step ($dt$). The code operates under these assumptions: (i) each particle starts with a mass ($m$) equal to the water depth in the cell where the particle is released. Since all injection cells have the same area (50 m × 50 m), it is equivalent to use the water depth or water volume in each cell. This initialization allows us to release a homogeneous concentration of pollutants in the GTM estuary. For example, if particles are injected into two cells with depths of 0.1 m and 1.2 m, their initial masses will be 0.1 and 1.2, respectively, so that the concentration is the same. We verified that the difference between the results obtained by using this method, and by injecting particles with same mass^31^ is negligible; (ii) oyster reefs gradually reduce the mass of suspended particles at each time step, proportionally to $F{R}_{R}$; (iii) particles are exclusively filtered when they pass over a reef; (iv) there is no increase in particle mass beyond the initial value; (v) particles are neutrally buoyant (i.e., do not settle or resuspend).

The material removed by the oysters populating a reef ($dm$) due to oyster filtration is:

4 $$dm=-\frac{m}{V} dt\,F{R}_{R},$$

where $V$ is the volume of water above the reef. Using the reef properties and the water depth at any time step $k$, Eq. (4) is used to calculate the fractional change (${F}_{j,i,k}$) in the mass of the ${i}^{th}$ particle across the ${j}^{th}$ reef, at that time step. Given the mass ${m}_{i,k}$ of the ${i}^{th}$ particle at the beginning of the time step $k,$ and knowing that the particle floats over the reef $j$ during that time step, at the start of the next time step, the mass is:

5 $${m}_{i,k+1}= {F}_{j,i,k}\,{m}_{i,k},$$

The MATLAB code tracks the quantity of particle mass cleared by each oyster reef at each time step. This enables the calculation of the overall particle mass removed from the estuary by each reef. Note that, in our approach, $FS$ is a real value ranging from 0 to 1, where a value of 1 indicates that 100% of the particle mass in the estuary is filtered by the oyster reefs in an estuary residence time. Finally, to assess the individual contribution of each reef to ${FS}_{E}$ independent on the total mass of the oysters populating the reef, we calculate the ${FS}_{R}$ values per unit of oyster mass (${FS}_{R}^{{M}_{O}}$).

### Filtration services scenarios

To understand how bank erosion affects estuarine ecosystems, we run various simulations. In each simulation, we gradually remove oyster reefs located at different distances from the ICW bank, to mimic the impact of bank erosion on the survival of the reefs, and we recalculate the estuarine filtration services by considering only the remaining reefs. In the simulated scenarios, bank erosion ranges from 0 to 1200 m from the current bank, with a difference of 30 m between consecutive simulations. In total, we run 40 scenarios.

### Supplementary Information

Supplementary Figures.

## Data Availability

All data, code, and materials used in the analyses will be available upon reasonable request. To request data, please contact the corresponding author, Daniele Pinton (daniele.pinton@ufl.edu), or the contributing author, Alberto Canestrelli (alberto.canestrelli@essie.ufl.edu).
