# Supplementary material for: Bank erosion drastically reduces oyster reef filtration services in estuarine environments
Source: Sci Rep. 2024 Jul 9;14:15812. doi: 10.1038/s41598-024-66670-1 (PMC11233518; doi:10.1038/s41598-024-66670-1)
Supplement: Supplementary file 1 — Supplementary Figures. [file 41598_2024_66670_MOESM1_ESM.docx]

**Bank Erosion Drastically Reduces Oyster Reef Filtration Services in Estuarine Environments**

**Daniele Pinton,^1^* Alberto Canestrelli^1^**

1. Department of Civil and Coastal Engineering, University of Florida, Gainesville, Florida, United States.

*Corresponding author: Daniele Pinton, +1 352-246-5839, [daniele.pinton@ufl.edu](mailto:daniele.pinton@ufl.edu).

# Supplementary Information


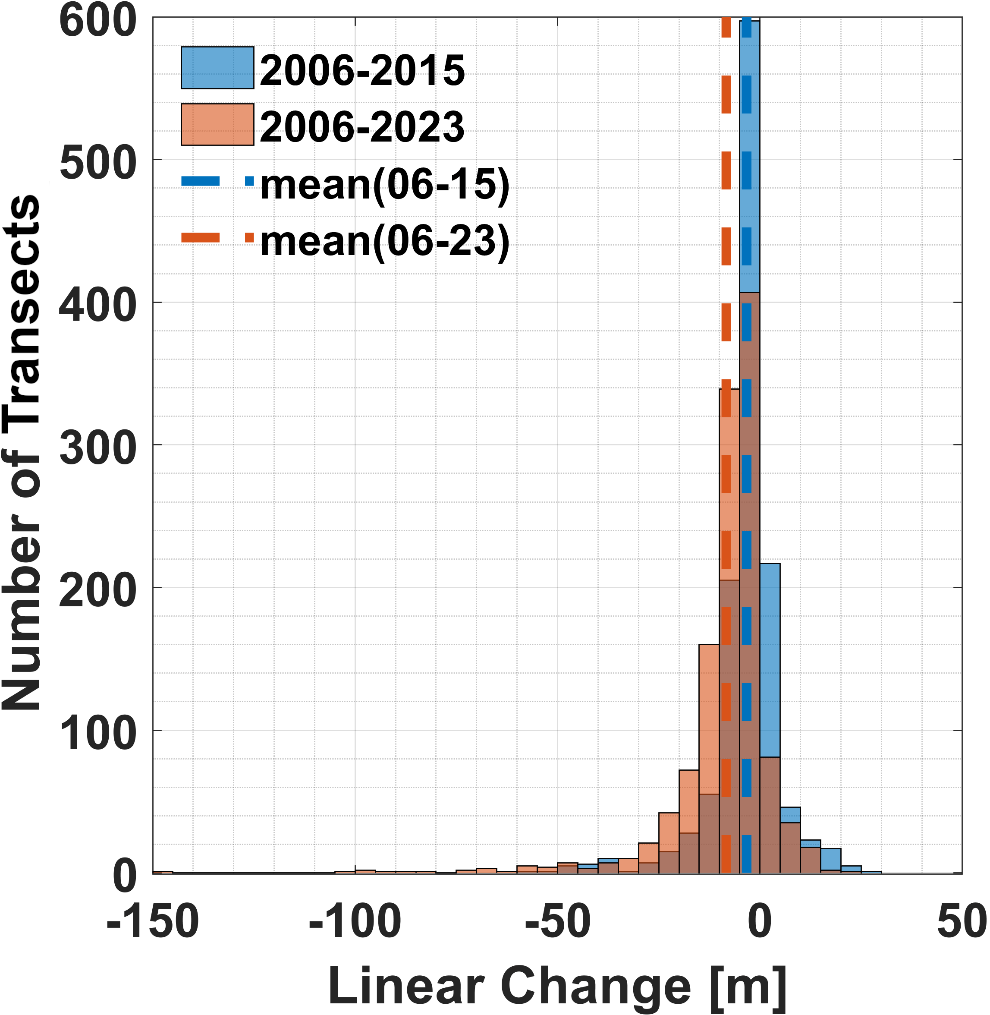


**Figure S1.** Bank erosion estimate. The distribution of lateral bank movement in the ICW measured for the periods 2006-2015 (blue bars) and 2006-2023 (orange bars) using historical airborne images. The bin size is 5 meters. The blue and orange dashed lines represent the average bank erosion estimated for the respective time periods.

**Error Analysis on Bank Erosion Estimates**

The total error in identifying the marsh edge is considered as the sum of the maximum horizontal accuracy value of the imagery datasets and the systematic error in the manual identification of the marsh bank.

The horizontal accuracy of the imagery datasets from the NOAA database for the years 2006, 2015, and 2023 are 5 m, 1.5 m, and 5 m, respectively, according to the metadata in the NOAA inventory (<https://www.coast.noaa.gov/dataviewer/#/>). The systematic error due to the manual identification error for the marsh edge (last vegetated cell) is assumed to be 1 pixel. Given that the pixel dimensions are 0.5 m for both 2006 and 2015, and 0.3 m for 2023, we assume a systematic error of 0.5 m. Therefore, the total error in identifying the marsh edge is equal to ±5.5 m.

After accounting for this error in the bank erosion estimates from imagery datasets, the average bank erosion rate from 2006 to 2023 ranges from -0.16 to -0.81 meters per year. Thus, the projected reduction in $FS$ over 100 years, based on these erosion rates, ranges from 8% to 32%”.


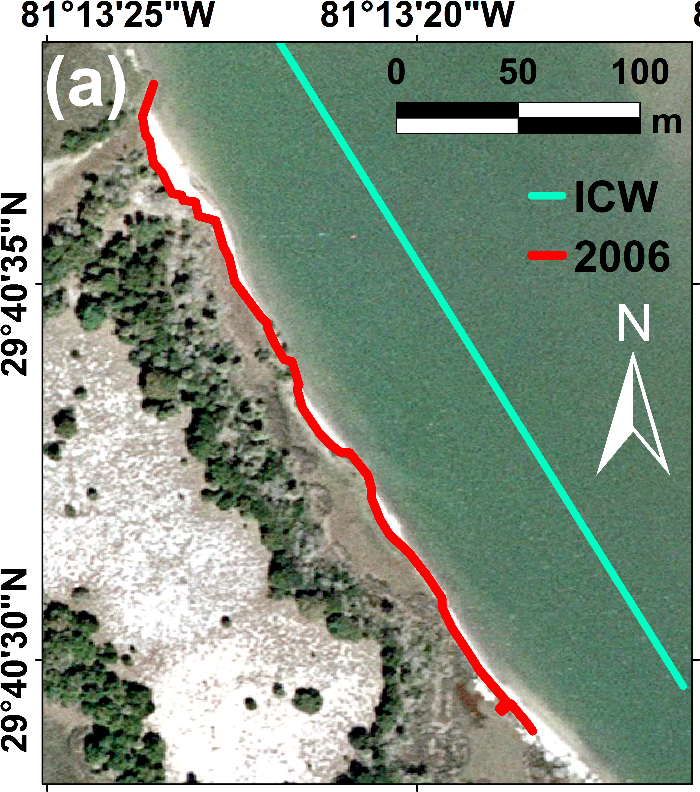

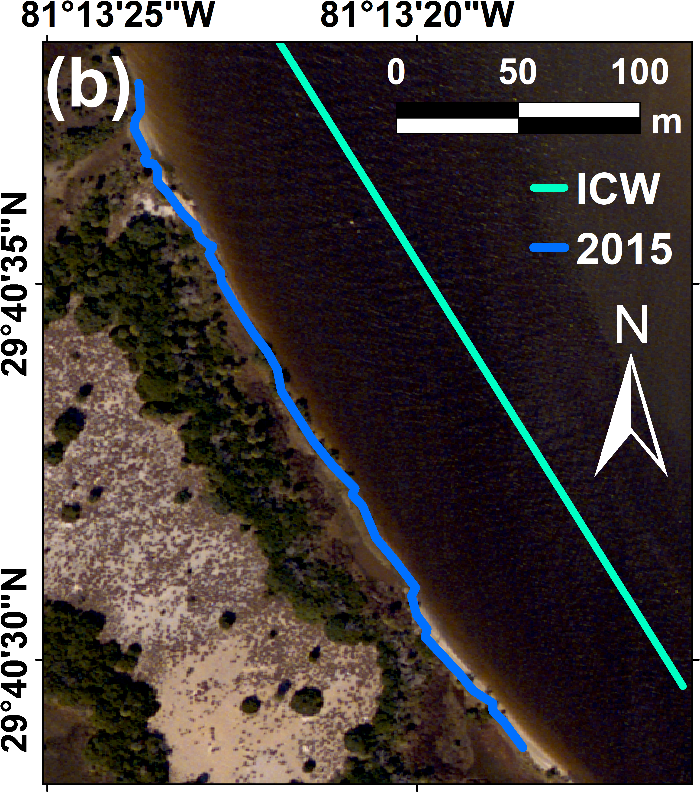


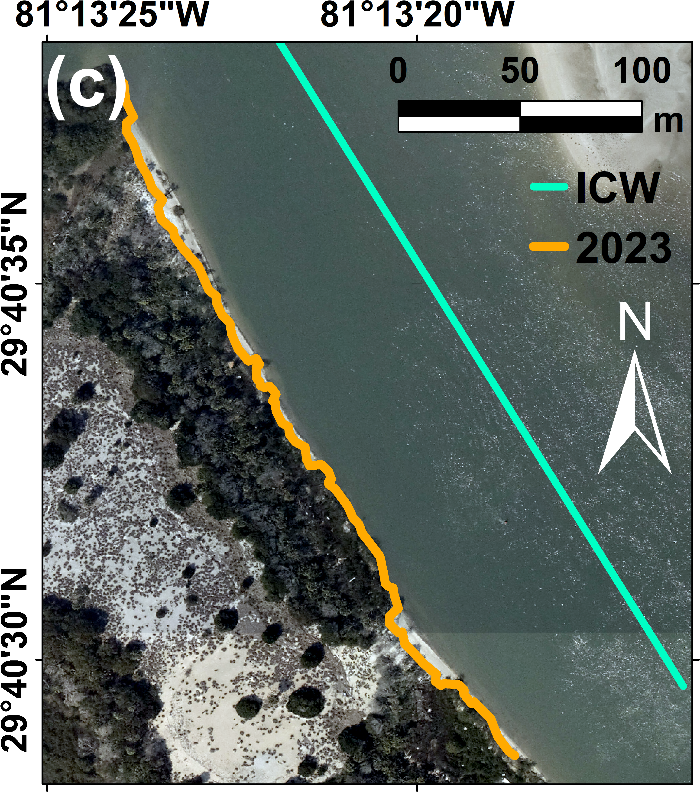

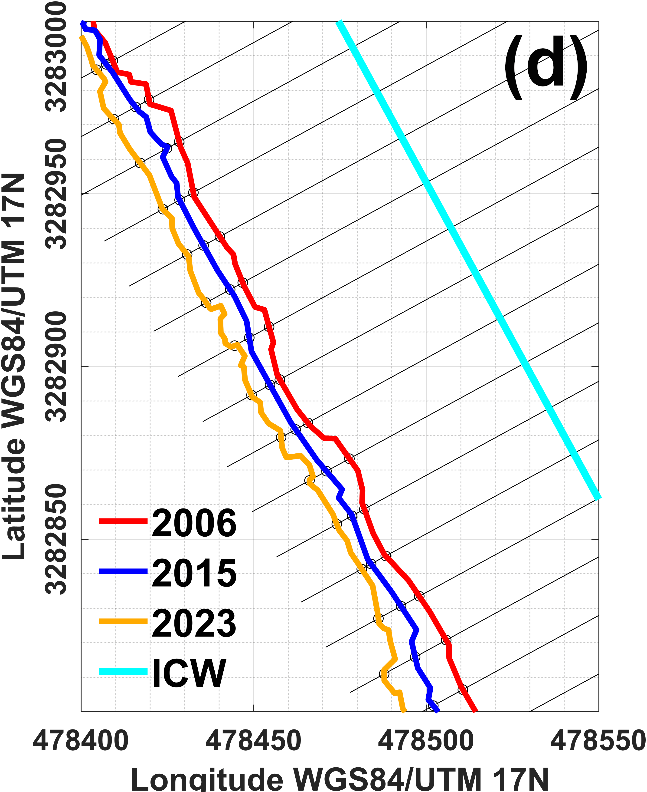


**Figure S2**. Visualization of the extraction process for marsh erosion in the GTM estuary. (**a-c**) Example of the salt marsh edges derived from the 2006 (**a**), 2015 (**b**), and 2023 (**c**) imagery datasets in the GTM estuary. (**d**) Example of the segments, perpendicular to the Intracoastal Waterway centerline, intersecting the marsh edges and used to evaluate bank erosion. (Maps generated using ESRI ArcGIS, v.10.8.1, <https://www.arcgis.com/index.html>).


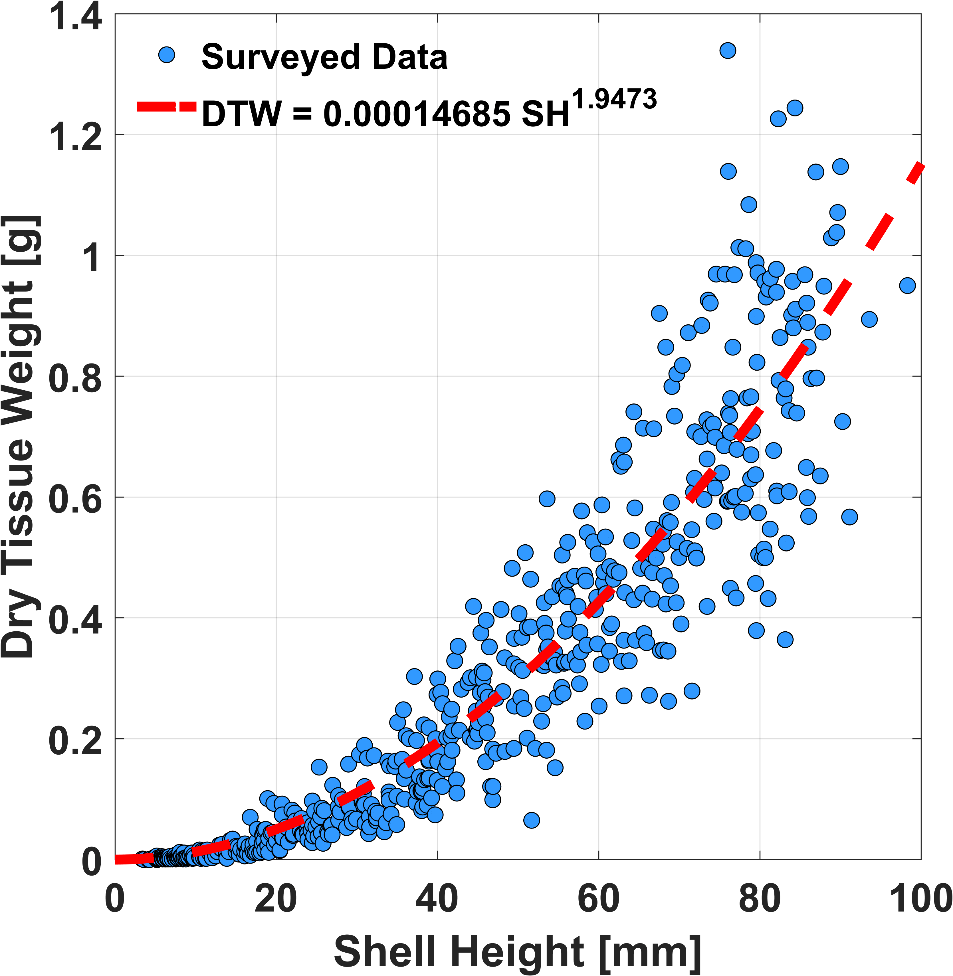


**Figure S3.** The blue dots represent the dry tissue weight ($DTW$) as a function of shell height ($SH$) on the $y$-axis and $x$-axis, respectively. This data was obtained from field surveys performed in the GTM estuary, as reported by Gray et al.^1^. Surveys had been conducted at seven different stations distributed throughout the estuary. The red dashed line indicates the allometric function fitting the surveyed data.


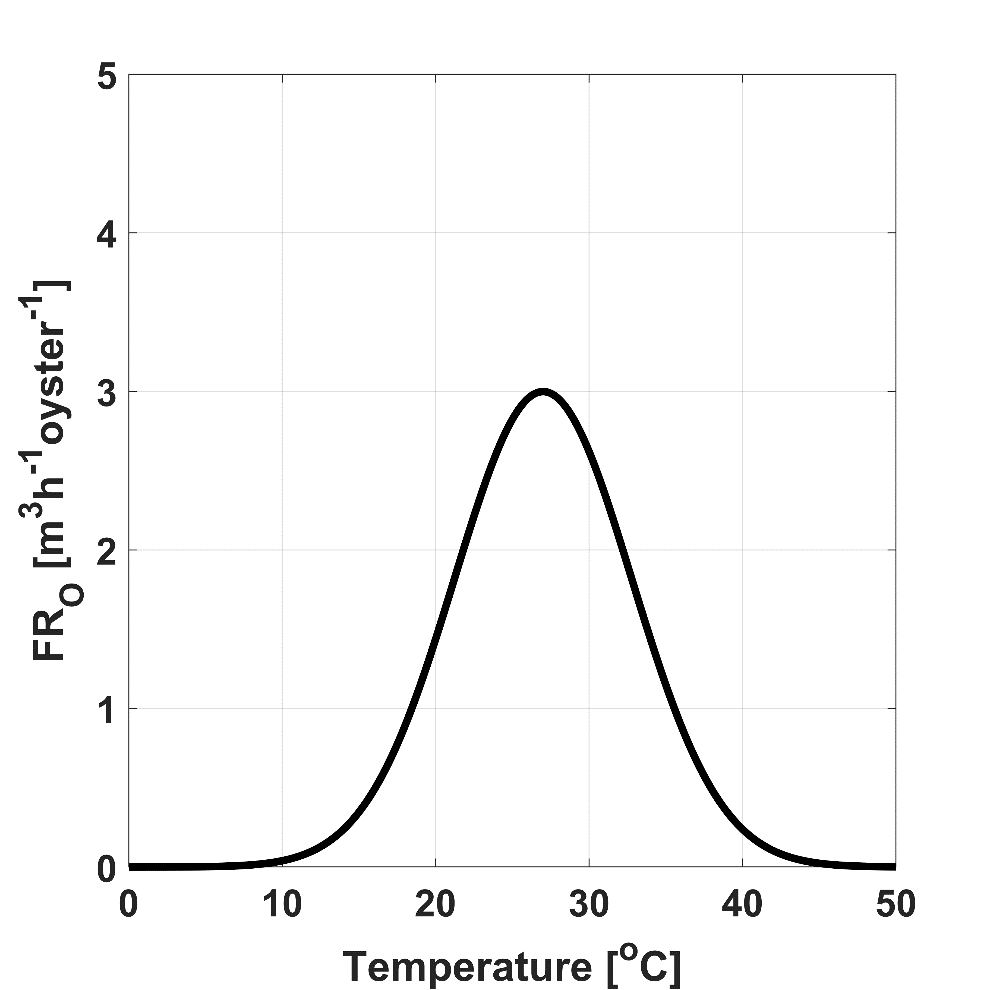


**Figure S4.** The Filtration Rate of an individual oyster (measured in m^3^h^-1^oyster^-1^) obtained from Equation (3) in the manuscript is shown for varying water temperatures ($x$-axis) and for the dry mass ($DTW$) of the average oyster in the GTM estuary ($y$-axis).

# References

1. Gray, M. W. *et al.* Beyond Residence Time: Quantifying Factors that Drive the Spatially Explicit Filtration Services of an Abundant Native Oyster Population. *Estuaries and Coasts* **45**, 1343–1360 (2021).
